# Accuracy of MRI in early rectal cancer: national cohort study

**DOI:** 10.1093/bjs/znac059

**Published:** 2022-03-12

**Authors:** Roberto Rosén, Emelie Nilsson, Milladur Rahman, Carl-Fredrik Rönnow

**Affiliations:** Department of Clinical Sciences, Malmö, Section of Surgery, Skåne University Hospital, Lund University, Malmö, Sweden; Department of Clinical Sciences, Malmö, Section of Surgery, Skåne University Hospital, Lund University, Malmö, Sweden; Department of Clinical Sciences, Malmö, Section of Surgery, Skåne University Hospital, Lund University, Malmö, Sweden; Department of Clinical Sciences, Malmö, Section of Surgery, Skåne University Hospital, Lund University, Malmö, Sweden

## Abstract

MRI plays a pivotal role in the staging of early rectal cancer, resulting in allocation of patients to surgery or organ-sparing treatment. In this large population-based retrospective cohort study, MRI substantially understaged pT3 and overstaged pT1 rectal cancer, in addition to unreliable nodal staging. Based on these findings, MRI is not adequate in allocating patients with rectal cancer to organ-sparing treatment.

## Introduction

Radiological staging of rectal cancer dictates subsequent patient treatment. In early-stage disease, local excision is associated with reduced morbidity, mortality, and costs, and maintains bowel continuity compared with surgery, where the whole or part of the rectum is resected^1–3^. Nearly 90 per cent of patients with T1 rectal cancer have N0 disease and are therefore potentially curable with local resection, yet the majority undergo major resection^4–6^. MRI is the primary staging investigation used to predict local stage in rectal cancer^7^, mainly owing to its ability to allocate patients in need of neoadjuvant treatment^8–10^. There is potentially inaccuracy in MRI staging for nodal involvement and differentiation of T1 from T2 tumours^6,7,11^. Consequently, cT1 and cT2 are often combined and comprice tumours considered for local resection. Apart from a recent study^6^ reporting 54 per cent accuracy for MRI cT1–2 category, combined cT1–2 status has not been investigated.

The aim of this large nationwide retrospective cohort study was to investigate the staging accuracy of MRI, from a clinical perspective, in early rectal cancer when combining cT1 and cT2 categories.

## Methods

### Patients

This study was approved by the Regional Ethical Review Board, Lund University (2017/546). All patients undergoing surgical resection for rectal cancer between 2009 and 2018 whose tumours were categorized before surgery as cT1–T2 (primary cohort), and all those with pT1 disease (secondary cohort) were identified from the prospectively maintained Swedish Colorectal Cancer Registry (SCRCR). Exclusion criteria were lack of MRI, neoadjuvant treatment, emergency resection, missing pathological T or N category, and time to surgery after MRI exceeding 1 year.

### Staging accuracy and statistics

Clinical MRI-based cT1–2 and cN categories were compared with the final pathology. T category accuracy was measured as positive predictive value (PPV). Specificity, sensitivity, PPV, negative predictive value, positive likelihood ratio, and negative likelihood ratio were calculated for lymph node category. Age, sex, time to surgery, year of operation, additional endoscopic ultrasonography (EUS), and hospital volume (low (centres treating fewer than 30 patients with MRI cT1–2 disease) *versus* high (over 30 patients with MRI cT1–2 disease)) were tested for possible impact on cT1–2 and cN accuracy in univariable and multivariable logistic regression analyses. *P* < 0.050 was considered significant.

Details of the SCRCR, preoperative staging, exclusion, staging accuracy, and statistics are shown in *Fig S1* and *Appendix S1*.

## Results

### Accuracy of T and N assignment in MRI cT1–2 cohort

In total, 1888 patients with MRI cT1–2 tumours, who had a median age of 71 years (42.8 per cent women), remained for analysis after excluding 407 patients (*Fig. S1*). Baseline characteristics are shown in *Table S1*. PPV for the combined MRI cT1–2 category was 67.8 per cent. Of the 1888 patients, 566 (30.0 per cent) and 41 (2.2 per cent) had pT3 and pT4 disease respectively (*Table 1*). Univariable and multivariable analysis identified low age, female sex, increasing time to surgery, EUS, and year of diagnosis (2016) as significant factors improving cT1–2 accuracy (*Table S2*). The PPV increased from 67.8 per cent to 85.2 per cent when EUS was performed.

**Table 1:** Tumour and nodal categories in MRI cT1–2 cohort

Pathological category				
**Clinical tumour category**	**pT1**	**pT2**	**pT3**	**pT4**
cT1–2	426	855	566	41
**Clinical node category**	**pN0**	**pN+**	**Total**	
cN0	1232	354	1586	
cN+	131	102	233	
cNx	48	21	69	
Total	1411	477	1888	
**Node category accuracy**				
Sensitivity (%)	21.4	Specificity (%)	87.3	
PPV (%)	43.8	NPV (%)	77.7	
LR+	1.69	LR–	0.90	

The analysis included 1888 patients. PPV, positive predictive value; NPV, negative predictive value; LR+, positive likelihood ratio; LR–, negative likelihoood ratio.

The accuracy, sensitivity, and specificity of MRI in detecting lymph node metastases in the cT1–2 cohort were 70.7, 21.4, and 87.3 per cent respectively (*Table 1*). MRI erroneously staged 354 pN+ tumours (74.2 per cent) as cN0, and 131 MRI cN+ tumours (56.2 per cent) were in fact pN0 (*Table 1*). Univariable and multivariable analysis identified diagnosis years 2010 and 2011 as the only significant factors influencing cN accuracy (*Table S3*).

### MRI accuracy in the pT1 cohort

After exclusion, 549 patients with a median age of 69 years (45.0 per cent women) remained for analysis from the 1846 patients identified as having pT1 rectal cancer (*Fig. S2*). Baseline characteristics are shown in *Table S4*. MRI assigned the tumour category erroneously in 123 (22.4 per cent) of 549 patients with pT1 disease (*Table 2*). MRI wrongly categorized 44 (70 per cent) of 63 pN+ tumours as cN0, and 38 (70 per cent) of the cN+ tumours were in fact pN0 (*Table 2*). The accuracy, sensitivity, and specificity of MRI for N category in pT1 rectal cancer was 81.1, 28.6, and 87.9 per cent respectively (*Table 2*).

**Table 2:** Tumour and nodal categories in pT1 cohort

Clinical category				
**Pathological tumour category**	**cT1–2**	**cT3**	**cT4**	**cTx**
pT1	426	67	3	53
**Pathological node category**	**cN0**	**cN+**	**cNx**	**Total**
pN0	427	38	21	486
pN+	44	18	1	63
Total	471	56	22	549
**Node category accuracy**				
Sensitivity (%)	28.6	Specificity (%)	87.9	
LR+	2.36	LR–	0.81	

The analysis included 549 patients. LR+, positive likelihood ratio; LR–, negative likelihoood ratio.

### MRI in patients eligible for local resection

MRI staged the disease in 1586 patients as cT1–2 N0, and this was correct in 933 (58.8 per cent). Thus, 653 MRI cT1–2 N0 (41.2 per cent) tumours were staged inaccurately. Concurrently, MRI overstaged 142 (29.2 per cent) of the 486 pT1 N0 tumours as either cT3–4 and/or cN+, potentially excluding local resection as a treatment option for these patients.

## Discussion

This population-based retrospective cohort study investigated the TN staging accuracy of MRI in early rectal cancer from a clinical perspective. Accuracy for the combined MRI cT1–2 category was 67.9 per cent, which is at the lower end of the range of 65–86 per cent for T category accuracy reported in previous studies^9,12–14^. The comparatively low accuracy here is somewhat surprising, considering that broadly defining cT1–2 (as opposed to T1 and T2 separately) was expected to increase diagnostic precision. However, MRI substantially understaged pT3 tumours in the present study, which constituted 30.0 per cent of all cT1–2 disease included. These findings are supported by a recent study by Detering and colleagues^6^, who reported accuracies of 70, 50, and 54 per cent for cT1, cT2, and cT1–2 respectively. Interestingly, previous studies suggested that most MRI staging failures occur when differentiating T2 and T3, and that these are caused by microscopic invasion of mesorectal fat^15^ and desmoplastic reactions surrounding lesions^9^ that are hard to distinguish as benign or malignant on MRI. Notably, additional EUS slightly improved cT1–2 accuracy, although the small number of patients in the present study hampers any conclusion.

The feasibility of local resection is not only dependent on cT category but also cN status^16^. MRI erroneously staged the majority of pN+ disease (74.2 per cent) as cN0 and, conversely, the majority of cN+ tumours (56.2 per cent) were pN0 in the present study. These findings are within the wide range of previous studies reporting N status accuracy of 43–85 per cent^6,13,14,17,18^. This large variation in the literature could possibly be explained by previous lack of consensus on size cut-off and morphological criteria used^7^.

Numerous factors are important when considering local resection, including tumour size and morphology as well as location, and additional investigations can be performed in unclear cases. It is generally accepted that local resection of rectal cancer should be considered only if the primary MRI investigation designates lesions as cT1–2 N0. Although the combined MRI cT1–2 category eliminates overstaging of pT1 as pT2, substantial overstaging of pT1 N0 lesions as cT3–4 and/or cN+ was noted in the present study. Thus, local resection was potentially excluded as an option by MRI in one-third of eligible patients. Concomitantly, combining cT1–2 led to substantial understaging, and in 41.2 per cent of patients disease staged as cT1–2 N0 was either pT3/4 and/or pN+. Taken together, the unreliability of MRI in staging early rectal cancer is a major concern when considering a patient for local resection.

The staging difficulties observed in the present study are of concern with respect to the use of (chemo)radiotherapy in early rectal cancer to facilitate organ preservation, as this approach may potentially overtreat T1 disease that could safely be treated by local excision alone. In contrast, local resection of early rectal cancer has the benefit of yielding exact histopathology, which is vital for assessing the risks of lymph node metastases and recurrence^5,16^. Further treatment, ranging from surveillance to salvage surgery and adjuvant chemoradiotherapy (within current trials^19^), could then be discussed accurately with the patient.

The present study is strengthened by the large population-based sample size and prospectively collected data. It is limited by not including patients with benign disease that was potentially overstaged by MRI as rectal cancer, and lack of standardization as well as information on MRI equipment and protocols.

This study found that MRI cT1–2 category is inadequate in identifying early rectal cancer eligible for local resection, with a substantial risk of understaging pT3 lesions and overstaging pT1 lesions, in addition to poor nodal accuracy. Thus, MRI alone should not dictate eligibility for local resection, which requires a multimodal approach by experienced clinicians.

## Supplementary Material

znac059_Supplementary_Data
