# Supplementary material for: Accuracy of MRI in early rectal cancer: national cohort study
Source: Br J Surg. 2022 Mar 12;109(7):570–2. doi: 10.1093/bjs/znac059 (PMC10364750; doi:10.1093/bjs/znac059)
Supplement: znac059_Supplementary_Data [file znac059_supplementary_data.zip › Supplementary_material.docx]

Supplementary material

*Patients*

All data was retrieved from the Swedish Colorectal Cancer Registry (SCRCR), a national quality registry containing prospectively collected data on preoperative staging, operative surgical details, histopathology, oncologic treatment and follow-up (5 years). The SCRCR coverage compared to the compulsory Swedish Cancer Registry was 99% for RC during the study period (2009-2018).

All patients undergoing surgical resection for RC between 2009 and 2018, preoperatively staged as cT1-T2 were identified in the SCRCR. We also identified a secondary cohort including all patients undergoing surgical resection for pT1 RC between 2009 and 2018, to capture potential erroneous staging of pT1 lesions. The following exclusion criteria were applied to both the cT1-2 and pT1 cohorts: missing preoperative MRI, neoadjuvant treatment, emergency surgical resection, pNx, missing data on preoperative or pathological T or N stages as well as time to surgery from MRI exceeding one year.

*Preoperative staging*

The standard preoperative work up of RC in Sweden comprise MRI, computed tomography of the chest and abdomen, colonoscopy and rigid recto/proctoscopy. Additional EUS is optional and subject to local traditions and availability. Clinical T and N stages reported in the SCRCR and herein are based on the second assessment of MRI images, performed by an experienced gastrointestinal radiologist prior to a multidisciplinary team conference. Possible dis-agreements between the first and second assessment are not reported in the SCRCR. All T and N stages are reported in the SCRCR following Swedish and European guidelines for colorectal cancer implementing the latest edition of the Union for International Cancer Control’s TNM classification. The assessment of lymph nodes recommended by the Swedish society of radiology are coherent to European guidelines and nodal metastases should be considered in lymph nodes ≥ 9mm in axial diameter or in lymph nodes 5-8 mm in axial diameter with at least two of three malignant morphological characteristics (round shape, irregular border or heterogenous signal)^8^. Only histopathologically verified RC are registered in the SCRCR and non-malignant tumours, preoperatively staged as cancers, are not registered and hence not included in this study.

*Staging accuracy*

The clinical T- and N- stages, investigated with MRI was compared with the pathologic T- and N- stages. MRI T-stages 1 and 2 are combined in the SCRCR (cT1-2), accurate when pT1 or pT2 were found in the specimen. Clinical and pathological nodal status were categorized as either N0 or N+, the latter comprising both N1 and N2. Accuracy was measured and presented as positive predictive value (PPV) for the MRI cT1-2 cohort. Specificity, sensitivity, PPV, negative predictive value (NPV), positive likelihood ratio (LR+) and negative likelihood ratio (LR-) were calculated for the ability to stage lymph nodes in the cT1-2 and pT1 cohorts. The following factors were tested for possible impact on cT1-2 and cN stage accuracy: age, sex, time to operation, year of operation, additional EUS and low vs high volume centres. The median number of MRI cT1-2 performed in the 52 contributing centers were 30. Low volume centers were defined as <30 cases and high volume centers >30 cases. These numbers account for cT1-2 staged cases only and is assumed to mirror the total volume of RC staging in each center.

*Statistical analyses*

Univariate logistic regression was used to investigate the relationship between the aforementioned factors and T and N staging accuracy of MRI. Multivariate logistic regression including the same variables was used to adjust for potential confounding factors. R programming language and R Studio (R core team 2020, R foundation for statistical computing, Vienna, Austria) were used for the statistical analyses. *P*-values < 0,05 were considered significant. Data are presented as median and range.

Table S1. Characteristics of MRI cT1-2 cohort

| Cases staged as cT1-2 | | 1888 |  |  |
| --- | --- | --- | --- | --- |
| Male |  | 1079 (57.2 %) |  |  |
| Female |  | 809 (42.8 %) |  |  |
| Age at diagnosis, years | | 71 (25-95) |  |  |
| Time to surgery, days | | 47 (1-362) |  |  |
| MRI centres (n 52) | |  |  |  |
| Low volume* (n 26) | | 280 (14.8 %) |  |  |
| High volume** (n 26) | | 1608 (85.2 %) |  |  |
| EUS | | 54 (2.9 %) |  |  |
| Surgery | |  |  |  |
| Anterior resection | | 1228 (65.0 %) |  |  |
| Rectum amputation | | 368 (19.5 %) |  |  |
| Hartman procedure | | 222 (11.8 %) |  |  |
| Other | | 70 (3.7 %) |  |  |
| Nodal status | |  |  |  |
| pN0 | | 1411 (74.7 %) |  |  |
| pN+ | | 477 (25.2 %) |  |  |

*Low volume centres defined as <30 MRI cases. **High volume centres

defined as >30 MRI cases

Table S2. Factors potentially influencing tumour staging in patients staged by MRI as cT1-2.

|  |  |  | Univariate | | |  | Multivariate | | |
| --- | --- | --- | --- | --- | --- | --- | --- | --- | --- |
|  |  |  | OR | 95% CI | *p*-value |  | OR | 95% CI | *p*-value |
|  |  |  |  |  |  |  |  |  |  |
| Age at diagnosis | (years) |  | 0.986 | 0.977-0.995 | < 0.01 |  | 0.986 | 0.977-0.995 | < 0.01 |
|  |  |  |  |  |  |  |  |  |  |
| Gender | Male |  | 1 | Ref. |  |  | 1 | Ref. |  |
|  | Female |  | 1.41 | 1.16-1.72 | < 0.001 |  | 1.47 | 1.200-1.796 | < 0.001 |
|  |  |  |  |  |  |  |  |  |  |
| Time to surgery | (days) |  | 1.003 | 1.000-1.006 | < 0.05 |  | 1.004 | 1.001-1.008 | < 0.01 |
|  |  |  |  |  |  |  |  |  |  |
| Year of surgery | 2009 |  | 1 | Ref. |  |  | 1 | Ref. |  |
|  | 2010 |  | 0.93 | 0.59-1.48 | 0.77 |  | 0.92 | 0.58-1.48 | 0.74 |
|  | 2011 |  | 0.70 | 0.45-1.10 | 0.12 |  | 0.67 | 0.43-1.06 | 0.09 |
|  | 2012 |  | 0.81 | 0.52-1.26 | 0.35 |  | 0.79 | 0.50-1.22 | 0.29 |
|  | 2013 |  | 1.33 | 0.85-2.09 | 0.21 |  | 1.33 | 0.84-2.11 | 0.22 |
|  | 2014 |  | 1.07 | 0.68-1.67 | 0.76 |  | 1.11 | 0.71-1.75 | 0.65 |
|  | 2015 |  | 1.20 | 0.77-1.86 | 0.42 |  | 1.20 | 0.77-1.89 | 0.42 |
|  | 2016 |  | 2.02 | 1.27-3.25 | < 0.01 |  | 2.23 | 1.39-3.60 | < 0.001 |
|  | 2017 |  | 1.06 | 0.69-1.61 | 0.79 |  | 1.14 | 0.74-1.75 | 0.55 |
|  | 2018 |  | 1.03 | 0.67-1.57 | 0.89 |  | 1.08 | 0.70-1.66 | 0.71 |
|  |  |  |  |  |  |  |  |  |  |
| EUS use | No |  | 1 | Ref. |  |  | 1 | Ref. |  |
|  | Yes |  | 2.79 | 1.38-6.42 | < 0.01 |  | 2.90 | 1.41-6.75 | < 0.01 |
|  |  |  |  |  |  |  |  |  |  |
| Center volume | Low |  | 1 | Ref |  |  | 1 | Ref |  |
|  | High |  | 0.91 | 0.69-1.19 | 0.49 |  | 0.80 | 0.60-1.05 | 0.11 |
|  |  |  |  |  |  |  |  |  |  |

OR: odds ratio; CI: confidence interval; EUS: endoscopic ultrasound. * Low volume centres defined as MRI cT1-2 cases below median (30 cT1-2 cases/centre). ** High volume centres defined as MRI cT1-2 cases above median (30 cT1-2 cases/centre).

Table S3. Factors potentially influencing nodal staging accuracy in patients staged by MRI as cT1-2.

|  |  |  | Univariate | | |  | Multivariate | | |
| --- | --- | --- | --- | --- | --- | --- | --- | --- | --- |
|  |  |  | OR | 95% CI | *p*-value |  | OR | 95% CI | *p*-value |
|  |  |  |  |  |  |  |  |  |  |
| Age at diagnosis | (years) |  | 1.00 | 0.99-1.01 | 0.77 |  | 1.00 | 0.99-1.01 | 0.73 |
|  |  |  |  |  |  |  |  |  |  |
| Gender | Male |  | 1 | Ref. |  |  | 1 | Ref. |  |
|  | Female |  | 1.16 | 0.95-1.42 | 0.14 |  | 1.18 | 0.96-1.44 | 0.12 |
|  |  |  |  |  |  |  |  |  |  |
| Time to surgery | (days) |  | 0.999 | 0.997-1.002 | 0.67 |  | 1.00 | 0.997-1.003 | 0.96 |
|  |  |  |  |  |  |  |  |  |  |
| Year of surgery | 2009 |  | 1 | Ref. |  |  | 1 | Ref. |  |
|  | 2010 |  | 0.58 | 0.36-0.92 | < 0.05 |  | 0.58 | 0.36-0.93 | < 0.05 |
|  | 2011 |  | 0.60 | 0.37-0.95 | < 0.05 |  | 0.60 | 0.37-0.96 | < 0.05 |
|  | 2012 |  | 0.65 | 0.41-1.02 | 0.07 |  | 0.65 | 0.41-1.03 | 0.07 |
|  | 2013 |  | 0.92 | 0.57-1.47 | 0.73 |  | 0.93 | 0.58-1.49 | 0.77 |
|  | 2014 |  | 1.00 | 0.62-1.61 | 0.99 |  | 1.02 | 0.63-1.64 | 0.95 |
|  | 2015 |  | 0.77 | 0.49-1.22 | 0.27 |  | 0.78 | 0.49-1.23 | 0.28 |
|  | 2016 |  | 1.28 | 0.79-2.07 | 0.31 |  | 1.32 | 0.81-2.13 | 0.26 |
|  | 2017 |  | 0.96 | 0.61-1.50 | 0.86 |  | 0.99 | 0.63-1.55 | 0.96 |
|  | 2018 |  | 0.92 | 0.58-1.44 | 0.72 |  | 0.93 | 0.59-1.46 | 0.76 |
|  |  |  |  |  |  |  |  |  |  |
| EUS use | No |  | 1 | Ref. |  |  | 1 | Ref. |  |
|  | Yes |  | 1.19 | 0.66-2.29 | 0.58 |  | 1.35 | 0.74-2.62 | 0.35 |
|  |  |  |  |  |  |  |  |  |  |
| Center volume | Low* |  | 1 | Ref |  |  | 1 | Ref |  |
|  | High** |  | 1.00 | 0.75-1.31 | 0.98 |  | 0.92 | 0.69-1.22 | 0.58 |
|  |  |  |  |  |  |  |  |  |  |

CI: confidence interval. OR: odds ratio. EUS: endoscopic ultrasound. ** High volume centres defined as MRI cT1-2 cases above median (30 cT1-2 cases/centre).

| Study population | | 549 |  |
| --- | --- | --- | --- |
| Male |  | 302 (55.0 %) |  |
| Female |  | 247 (45.0 %) |  |
| Age at diagnosis (years) | | 69 (31-94) |  |
| Time to surgery (days) | | 52 (1-362) |  |
| Surgery | |  |  |
| Anterior resection | | 352 (64.1 %) |  |
| Rectum amputation | | 103 (18.8 %) |  |
| Hartman procedure | | 46 (8.4 %) |  |
| Other | | 48 (8.7 %) |  |
| Nodal status | |  |  |
| pN0 | | 486 (88.5 %) |  |
| pN+ | | 63 (11.5 %) |  |

Table S4. Characteristics of pT1 cohort
