# Supplementary figures and images for: Accuracy of MRI in early rectal cancer: national cohort study
Source: Br J Surg. 2022 Mar 12;109(7):570–2. doi: 10.1093/bjs/znac059 (PMC10364750; doi:10.1093/bjs/znac059)

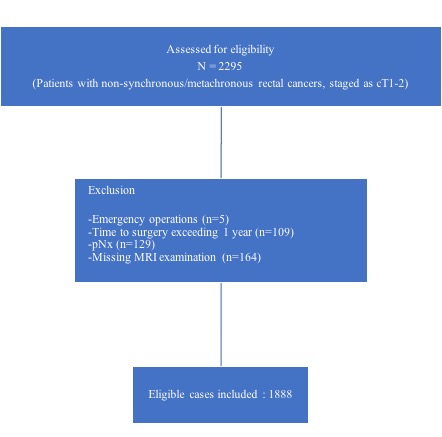

Supplement: znac059_Supplementary_Data [file znac059_supplementary_data.zip › Supplementary_Figure_1.jpg]

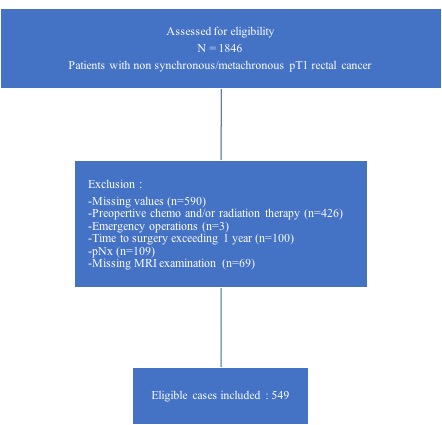

Supplement: znac059_Supplementary_Data [file znac059_supplementary_data.zip › Supplementary_Figure_2.jpg]
